# Retinoic acid inhibits the infection of porcine reproductive and respiratory syndrome virus

**DOI:** 10.3389/fvets.2026.1798441

**Published:** 2026-03-24

**Authors:** Jintong Guo, Shuangyi Xiehe, Mengmei Wang, Tianle Li, Qiyue Zheng, Ahui Wang, Shengcan Xie, Wenjing Wan, Yiyu Zhao, Wei Ma, Qin Zhao, Xin He, Young Tang

**Affiliations:** 1 Key Laboratory of Livestock Biology, Shaanxi Centre of Stem Cells Engineering & Technology, College of Veterinary Medicine, Northwest A&F University, Yangling, China; 2 Engineering Research Center of Efficient New Vaccines for Animals Ministry of Education, College of Veterinary Medicine, Northwest A&F University, Yangling, China

**Keywords:** inflammation, porcine alveolar macrophages, PRRSV, retinoic acid, RNA-Seq

## Abstract

**Introduction:**

Porcine reproductive and respiratory syndrome virus (PRRSV) is a major pathogen threatening the global swine industry, with existing vaccines failing to provide broad protection due to the high viral genetic variability and immune evasion capacity. Retinoic acid (RA), a vitamin A metabolite known for immunomodulatory functions, has been reported to enhance antiviral innate immunity and suppress excessive inflammation. However, whether RA affects PRRSV infection remains unclear.

**Methods:**

The antiviral efficacy of all-trans retinoic acid (ATRA), the major biologically active form of RA, against PRRSV infection was evaluated in both MARC-145 cells and primary porcine alveolar macrophages (PAMs). Furthermore, transcriptomic profiling was conducted to investigate the regulatory effects of ATRA on uninfected and PRRSV-infected PAMs.

**Results:**

ATRA significantly inhibited PRRSV infection and replication with minimal cytotoxicity. Transcriptomic profiling revealed that ATRA upregulated basal immune signaling and metabolic transport pathways while suppressing non-specific inflammatory activity in uninfected PAMs. Interestingly, comparative transcriptomics revealed that PRRSV infection led to hyperinflammatory responses and disrupted lipid homeostasis in PAMs, while ATRA treatment reversed these alterations.

**Discussion:**

Our findings elucidate that RA exerts antiviral effects against PRRSV through a dual mechanism of attenuating excessive inflammation and restoring cellular metabolic homeostasis, highlighting its potential as an immunomodulatory agent for viral infection control.

## Introduction

Porcine reproductive and respiratory syndrome (PRRS), a major viral infectious disease confronting the global swine industry, has caused sustained economic losses worldwide since its first report in the 1980s (1). Its etiological agent, PRRS Virus (PRRSV), is recognized as one of the core pathogens endangering the healthy development of swine industry (2). PRRSV possesses biological characteristics such as high genomic variability, serotype diversity, and strong immune escape ability (2), posing severe challenges to clinical prevention and control. Existing commercial vaccines, including inactivated and attenuated live vaccines cannot effectively cover all circulating strains, especially the emerging recombinant variants, and struggle to induce a fully protective cross-reactive immune response (3). PRRSV infection often leads to reproductive disorders in pregnant sows (e.g., abortion, stillbirth) and severe respiratory symptoms in piglets. Furthermore, it impairs the porcine immune system by interfering with interferon production and modulating cytokine expression, thereby increasing the risk of secondary infections by bacteria (e.g., *Haemophilus parasuis*, *Streptococcus* spp.) and other viruses (e.g., Porcine Circovirus Type 2) (4). This creates a complex pattern of coinfection and exacerbates PRRS control difficulty and economic losses (1).

Retinoic acid (RA), a vital metabolite derived from vitamin A, is a collective term for biologically active vitamin A derivatives that include all-trans retinoic acid (ATRA), 9-cis retinoic acid and 13-cis retinoic acid. Among these, all-trans retinoic acid (ATRA) is the most abundant and biologically active form of RA in mammalian cells and tissues, and is the primary ligand that activates the retinoic acid receptor (RAR) signaling pathway to exert immunomodulatory and antiviral effects (5). RA plays a central role in maintaining immune tolerance and restraining autoimmunity by directing T cell differentiation. It promotes the induction and stability of regulatory T cells (Tregs) via the retinoic acid receptor alpha (RARα) activation and synergizes with transforming growth factor-beta (TGF-β) to enhance forkhead box P3 (*Foxp3*) expression (6–8). Simultaneously, RA suppresses pro-inflammatory T helper 17 (Th17) and T helper 1 (Th1) cell differentiation by inhibiting key transcription factors such as retinoic acid-related orphan receptor *γ*t (RORγt) and reducing the production of cytokines like interleukin-17 (IL-17) and interferon-gamma (IFN-γ) (9–11). Furthermore, RA contributed to tissue-specific immune regulation by driving the expression of gut-homing receptors C-C motif chemokine receptor 9 (CCR9) and integrin α4β7, on T cells, thereby supporting mucosal tolerance (12). Its influence extends to innate immunity and B cell function, promoting anti-inflammatory macrophage phenotypes and IgA class switching, which collectively help prevent autoimmune pathology (12–14). RA enhances host innate immunity primarily by amplifying the Type I interferon (IFN) response through the activation of interferon regulatory factor 3 (IRF3) and calcium/calmodulin-dependent protein kinase kinase (CaMKK) signaling, thereby strengthening defense against viruses such as measles and parainfluenza (15–17). However, how RA signaling activity affects PRRSV infection is currently unknown. Given its well-documented immunomodulatory and antiviral properties, we hypothesized that RA signaling could effectively inhibit PRRSV infection by regulating host immune responses and restoring cellular metabolic homeostasis. To test this hypothesis, the primary objectives of this study were to: (1) evaluate the antiviral efficacy of ATRA against classical and highly pathogenic PRRSV strains in both MARC-145 cell lines and primary porcine alveolar macrophages (PAMs); and (2) elucidate the underlying molecular mechanisms by which ATRA modulates host immune and lipid metabolic responses during PRRSV infection using transcriptomic profiling. Here, we report that the activation of RA signaling by ATRA indeed leads to a significant inhibition of PRRSV infection.

## Materials and methods

### Chemicals, cells, viruses, and plasmids construction

PAMs were harvested from healthy, PRRSV-negative Landrace/Yorkshire cross pigs aged 4–6 weeks. Briefly, the pigs were slaughtered, and their lungs were transferred on ice to a cell culture cabinet. Warm phosphate-buffered saline (PBS) containing 200 U/mL penicillin and 200 μg/mL streptomycin was carefully injected through the trachea into the bronchi on both sides of the lungs. The lungs were then massaged, and bronchoalveolar lavage fluid (BALF) was retrieved. The BALF was centrifuged at 400 × *g* for 15 min, and the pellets were washed two times with warm complete medium. Cells were counted and frozen in 90% fetal bovine serum (FBS) and 10% dimethyl sulfoxide (DMSO) and stored in liquid nitrogen. PAMs were cultivated in RPMI-1640 supplemented with 10% FBS, 2 mM GlutaMAX Supplement, 0.1 mM minimum essential medium (MEM) non-essential amino acids, 1 mM sodium pyruvate, 100 U/mL penicillin, 100 μg/mL streptomycin, and 0.5 μg/mL Amphotericin B. The parental MARC-145 cell line used in this study was purchased from Stemrecell (Nanjing, China; https://www.stemrecell.com/cell-line-other-monkey/marc145.html, Cat. No. STM-CL-8145), which is derived from the fetal kidney epithelial cells of African green monkey (Chlorocebus aethiops) and is a clonal subline of the MA-104 cell line (ATCC® CRL-12231™, RRID: CVCL_4540). Pig CD163 (pCD163) was amplified from the pig genome and inserted into the pMXs retroviral vector (Cell Biolabs, CA, USA). To establish a pCD163-expressing MARC-145 cell line (pCD163-MARC-145) for the propagation and titration of PRRSV strains, a pCD163-expressing retrovirus was produced. Briefly, pMXs-pCD163 was co-transfected with pUMVC and pCMV-VSV-G (Addgene, MA, USA) packaging plasmids into HEK293T cells using Fugene 6 (Promega, WI, USA). Supernatants containing the virus were collected at 48 and 72 h post-transfection, following previously described protocols (18).

### Fluorescence-activated cell sorting

MARC-145 cells were cultivated in FP medium (Dulbecco’s Modified Eagle Medium (DMEM) containing 10% FBS, 2 mM GlutaMAX Supplement, 0.1 mM MEM non-essential amino acids, 50 U/mL penicillin, and 50 μg/mL streptomycin). These cells were then incubated with the pCD163-expressing retrovirus at 32 °C while being centrifuged at 650 × *g* for 45 min. The infection process was repeated after 24 h. Following the second infection, the cells were stained with CD163 Monoclonal Antibody (2A10/11), phycoerythrin (PE) (1:10, Thermo Fisher Scientific, MA, USA) at 24 h post-infection. Fluorescence-positive cells were sorted using an Attune NxT Flow Cytometer with a sorting module (Thermo Fisher Scientific, Waltham, MA, USA). All PRRSV strains were propagated and titrated in pCD163-MARC-145 cells, following previously described protocols (19, 20).

### PRRSV infection assay

PAMs were cultivated in 12-well plates and infected with the PRRSV SD-16 strain, VR-2332 strain, or JXA1-GFP strain at a multiplicity of infection MOI of 0.05 or 0.1 for 1 h. Cells were then incubated with 5 to 30 μM RA (retinoic acid) for 24 h at 37 °C. RNAs were extracted from the PAMs for reverse transcription quantitative-PCR (RT-qPCR) using RNeasy Minikits (Qiagen, MD, USA), and supernatants of cells were collected with PRRSV titration assay.

### PRRSV titration assay

pCD163-MARC145 cells were cultivated in 48-well plates and were inoculated with 10-fold serially diluted supernatants from PRRSV infection assay (6 wells for each dilution) at 37 °C for 2 h. The inoculum was replaced by DMEM supplemented with 2% FBS, 2 mM GlutaMAX supplement, 0.1 mM MEM non-essential amino acids, 50 U/mL and 50 μg/mL penicillin–streptomycin. Cells with cytopathic effect were recorded, and the median tissue culture infectious dose (TCID_50_/mL) was calculated using the Reed and Muench method.

### Cytotoxicity assay

The cytotoxicity of retinoic acid on porcine alveolar macrophages (PAM) was evaluated using the Cell Counting Kit-8 (CCK-8) assay. PAMs cultured in 24-well plates were treated with various concentrations of retinoic acid for 24 and 48 h, respectively. Following treatment, 100 μL of CCK-8 solution was added to each well, and the plates were incubated for an additional 2 h. Absorbance was measured at 450 nm using a microplate reader. The experiment included a DMSO vehicle control group and a blank control group for accurate assessment of the compound’s effect on cell viability.

### Reverse transcription quantitative-PCR

Total RNA from infected PAMs was reverse transcribed into cDNA using PrimeScript™ RT Reagent Kit with gDNA Eraser (Perfect Real Time) (Takara, Kyoto, Japan). The specific primers used for reverse transcription quantitative PCR (RT-qPCR) in this study are listed in Supplementary Table S13. RT-qPCR assays were performed on the Applied Biosystems (ABI) 7500 Fast Real-Time PCR System (Thermo Fisher Scientific, Waltham, MA, USA) with ChamQ SYBR qPCR Master Mix (Vazyme Biotech Co., Ltd., Nanjing, China; Cat. No. Q311-02). For normalization of total RNA input and correction of experimental variations, glyceraldehyde-3-phosphate dehydrogenase (GAPDH) was used as the reference gene for PAMs and MARC-145 cells. The expression stability of these reference genes across different viral infection and ATRA treatment conditions was verified prior to final analysis to ensure reliable normalization (21). Furthermore, the amplification efficiencies of all target and reference genes were evaluated using standard curves and confirmed to be approximately equal and near 100%. Therefore, relative transcript levels of target genes were calculated using the 2^−ΔΔCt^ (threshold cycle) method (22), and the results were presented as the fold change relative to the mock-treated control group.

### Immunofluorescence

PAMs were seeded in 12-well plates and inoculated with SD16 strains and VR2332 strains at MOI = 0.1 for 1 h. Following the removal of the virus inoculum, cells were treated with 5–20 μM ATRA or DMSO as a vehicle control for 24 h. The cells were then fixed with 4% paraformaldehyde for 15 min, permeabilized with 0.5% Triton X-100 for 15 min, and blocked with 5% bovine serum albumin in goat serum for 1 h at room temperature (RT). Subsequently, the cells were incubated with rabbit polyclonal antibodies against the PRRSV N protein (1:300, GeneTex, 16646-1-AP, CA, USA) for 2 h at RT. After washing with PBS, the cells were incubated with secondary antibodies conjugated to Fluorescein (FITC) (1:500, Proteintech, SA00003-2, Wuhan, China) for 1 h at RT. Finally, nuclei were counterstained using 4,6-diamidino-2-phenylindole (DAPI) (Sigma-Aldrich, MO, USA). Images of the cells were captured using the EVOS M7000 (Thermo Fisher Scientific). The percentage of PRRSV positive cells was calculated by dividing the number of green fluorescence-positive cells (PRRSV) by the number of blue fluorescence-positive cells (DAPI).

### Western blot

Protein samples were collected from mock control, PRRSV-infected control, and 20 μM ATRA-treated PRRSV-infected cells. Equal amounts of protein from cell lysates were separated by sodium dodecyl sulfate-polyacrylamide gel electrophoresis (SDS-PAGE) and transferred onto nitrocellulose membranes. The membranes were blocked with 5% non-fat milk in phosphate-buffered saline with Tween 20 (PBS-T) at room temperature for 1 h, followed by incubation with Anti-PRRS virus Nucleocapsid protein antibody (GTX129270; GeneTex, Irvine, CA) supernatant overnight. After three washes with PBS-T, the blots were incubated with (horseradish peroxidase) HRP-Goat Anti-Rabbit Recombinant Secondary Antibody (H + L-specific, 1:6,000; Proteintech, RGAR001) at room temperature for 1 h. Signals were detected using an Ultra High Sensitivity ECL Kit (MedChemExpress, HY-K1005, Monmouth Junction, NJ) and visualized with ChampChemi i610Plus. All immunoblots were performed in three biological replicates, and the data were processed and visualized using Image Lab and GraphPad Prism software.

### RNA sequencing and bioinformatics analysis

PAM cells were seeded in a 12-well plate and divided into the following groups: B (DMSO), D (DMSO + VR2332), RA_V (RA 10 μM + VR2332), RA (RA 10 μM). After 24 h, the cells were trypsinized, centrifuged, and washed two times with PBS. TRIzol was added, and the samples were scraped into centrifuge tubes, and total RNA was extracted. The quality of the samples was assessed using the Agilent 2100 Bioanalyzer, and transcriptome sequencing was performed on the Illumina sequencing platform in Novogene Co., Ltd. (Tianjin, China). The sequencing data were processed using a pipeline that included fastp, HISAT2, StringTie, and featureCounts to generate the expression matrix (RefSeq assembly accession: ensembl_104_sus_scrofa_sscrofa11_1_toplevel). After normalization, differential expression analysis was conducted using the DESeq2 (v1.20.0). The *p* values were adjusted using the Benjamini and Hochberg method, and adjusted p values ≤ 0.05 and |log_2_ (fold change)| ≥ 1 were set as the thresholds for significant differential expression. Gene Ontology (GO) and Kyoto Encyclopedia of Genes and Genomes (KEGG) enrichment analyses of differentially expressed genes were performed using the clusterProfiler software (v3.8.1). Alternative splicing event analysis was conducted using the rMATS (V4.1.0).

### Statistics analysis

All experimental data were first tested for normality using the Shapiro–Wilk test, and the results confirmed that the data conformed to a normal distribution (*p* > 0.05), meeting the application criteria for parametric statistical tests. Data were subsequently analyzed by one-way analysis of variance (ANOVA) with Tukey’s *post hoc* test for multiple group comparisons or two-tailed Student’s t-test for comparisons between two groups. All results are presented as the mean ± standard deviation (SD) of at least three independent biological replicates. A *p*-value < 0.05 was considered statistically significant. All statistical analyses were performed using GraphPad Prism software (Version 9.0, GraphPad Software, Inc., San Diego, CA, USA).

## Results

### ATRA inhibits PRRSV infection in MARC-145 cells

**Figure 1:**
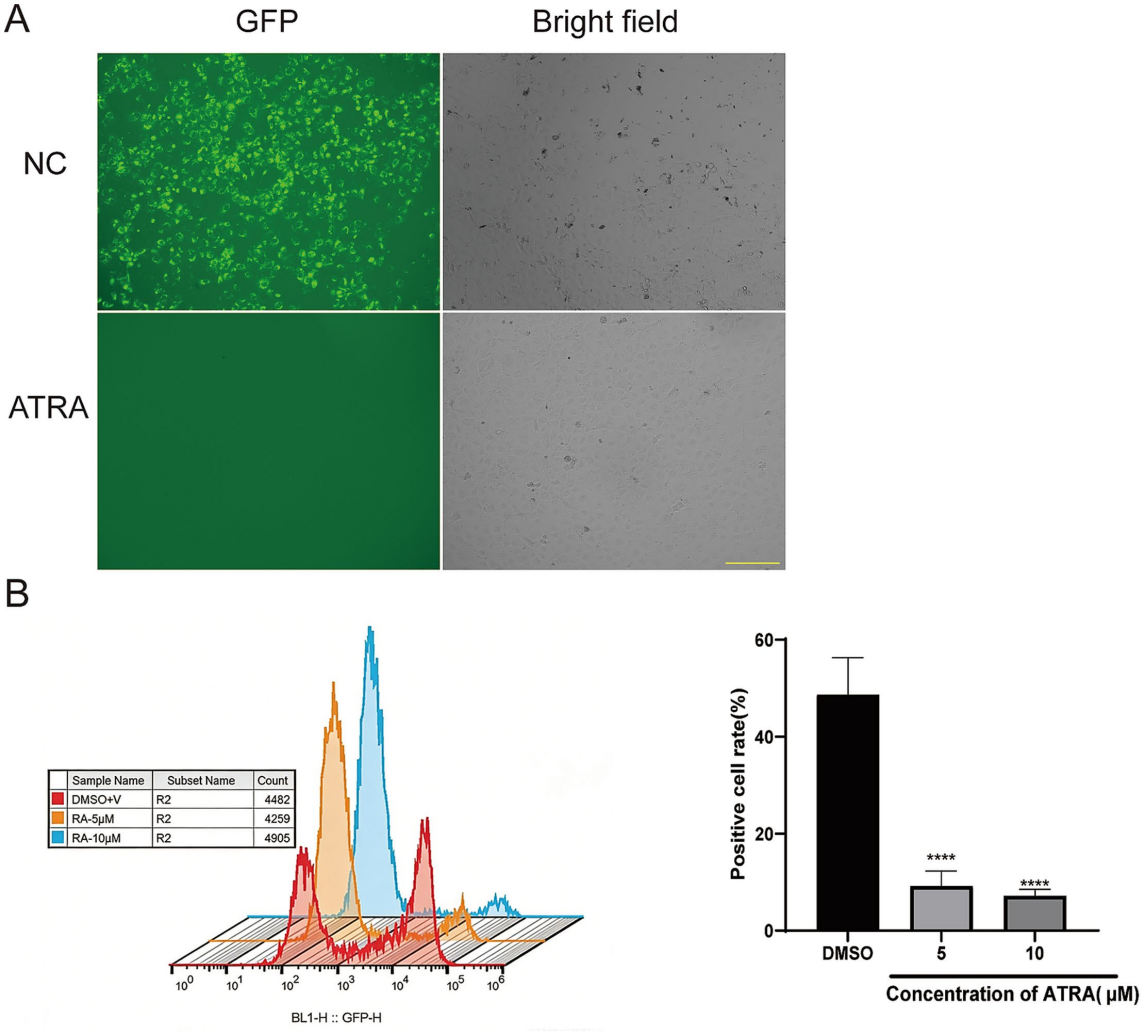
ATRA blocks PRRSV infection of MARC-145 cells. **(A)** In cell screening: fluorescence signal visualization of the negative control (NC) and ATRA-treated groups. Bar = 200 μm. **(B)** Left: FACS analysis of ATRA on PRRSV infection in MARC-145 cells, viral infection was evaluated via GFP fluorescence. Right: GFP positive cell rate was quantified for control group and groups receiving different concentrations of ATRA. Values represent mean ± SD, *n* = 3. ****p < 0.0001.

To investigate whether RA signaling activity affects PRRSV infection, the reverse-genetics system engineered GFP-positive JXA1 (JXA1-GFP) strain (23) with MOI of 0.05 was used to inoculate MARC-145 cells, a commonly used cell line for PRRSV amplification, followed by the addition of either DMSO as the vehicle control or 5–10 μM ATRA treatment. For the DMSO condition, viral GFP signal was readily observable under the fluorescence microscope at 48 h post-infection (hpi), while the PAMs under ATRA treatment exhibited little fluorescence with normal cell growth and morphology (Figure 1A). The cells were then collected and subjected to fluorescence-activated cell sorting (FACS). FACS analysis further revealed that compared with the control of near 50% viral-GFP positive (GFP+) cells, 5 and 10 μM ATRA treatments significantly reduced the GFP+ cell percentage to 12 and 7%, respectively (Figure 1B). Additionally, the propidium iodide (PI) staining of sorted cells identified that the viable cell percentage was consistently above 90% (Supplementary Figure S1), indicating a low cytotoxicity caused by ATRA treatment.

### Validation of the retinoic acid efficacy against PRRSV infection in pig cells

**Figure 2:**
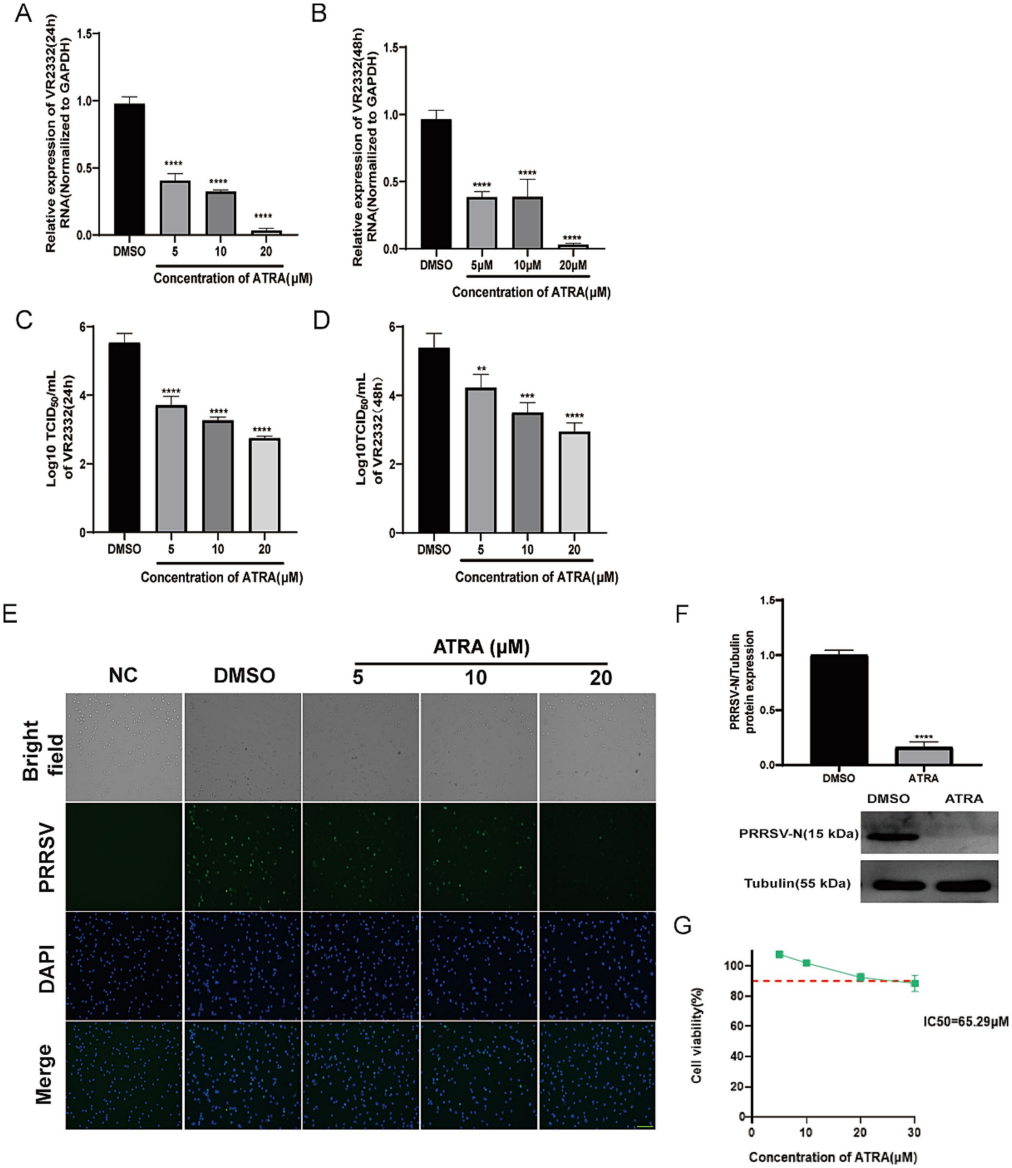
Validation of the retinoic acid efficacy against PRRSV infection in pig cells. **(A)** RT-qPCR analysis of VR2332 RNA in PAMs treated with different concentrations of ATRA (24 h). DMSO was used as negative controls. Values represent mean ± SD, *n* = 3. **(B)** RT-qPCR analysis of VR2332 RNA in PAMs treated with ATRA (48 h). All other conditions were identical to **(A)**. **(C)** Viral titration analysis in TCID_50_/mL for VR2332 in supernatants of PAMs treated as described in **(A)**. Values represent mean ± SD, *n* = 3. **(D)** Viral titration analysis in TCID_50_/mL for VR2332 in supernatants of PAMs treated as described in **(B)**. All other conditions were identical to **(C)**. **(E)** Representative immunofluorescence images of PAMs infected with or without VR2332 and treated with DMSO or ATRA at 24 h post-infection. NC: Non-infected cells treated with DMSO. DMSO: Infected cells treated with DMSO. Bar = 200 μM. **(F)** The antiviral effect of ATRA was evaluated by western blot analysis of VR2332 N protein levels. (Top) The relative protein expression of PRRSV-N was analyzed by western blotting and normalized to the loading control Tubulin. The bar graph shows the mean ± SD. ****p < 0.0001, *n* = 3. (Bottom) Representative western blot images of PRRSV-N (15 kDa) and Tubulin (55 kDa). **(G)** Cytotoxicity analysis of different concentrations of ATRA incubated with PAMs for 24 h, with DMSO as the vehicle control. The red dashed line indicates 90% relative viability. Values represent mean ± SD, *n* = 3. **p < 0.01, ***p < 0.001, ****p < 0.0001.

To further validate the antiviral function of ATRA, we investigated its effect on inhibition of PRRSV replication in porcine alveolar macrophages (PAMs) - the primary target cells by PRRSV in pigs. PAMs were inoculated with the type II PRRSV strain VR2332 with MOI of 0.1 for 2 h, followed by treatments with an ATRA gradient (5–20 μM). At 24 and 48 hpi, cells and culture supernatants were collected, with viral genome quantified by reverse transcription quantitative PCR (RT-qPCR) and viral titer determined via 50% tissue culture infectious dose (TCID_50_) assay. The RT-qPCR result identified that at both 24 and 48 hpi, 5–20 μM ATRA treatments led to a significant and dosage-responsive reduction of PRRSV RNA in PAMs compared with the DMSO control (Figures 2A,B). This was further validated by TCID_50_ assay, which revealed that compared with the control, there were decreased PRRSV titer of 1.5 (1), 2 (1.5), and 3 (2.5) log by 5, 10, and 20 μM ATRA treatments at 24 (48) hpi, respectively (Figures 2C,D). Immunofluorescence with an antibody against PRRSV nucleocapsid (N) protein confirmed that treatment with 5–20 μM ATRA significantly inhibited the infection of PRRSV in PAMs, with the viral signal nearly undetectable in 20 μM ATRA treatment (Figure 2E). Consistently, Western blot (WB) analysis targeting PRRSV N protein further verified that ATRA significantly reduced the expression level of PRRSV N protein in PAMs, which was barely detectable at 20 μM ATRA (Figure 2F). Furthermore, CCK-8 assay disclosed that the viability of PAMs remained above 90% after treatment with 1–30 μM ATRA for 48 h compared with the DMSO control, confirming that ATRA exhibited no significant cytotoxicity within the effective concentration range (Figure 2G).

### Retinoic acid inhibits highly pathogenic PRRSV strain infection

**Figure 3:**
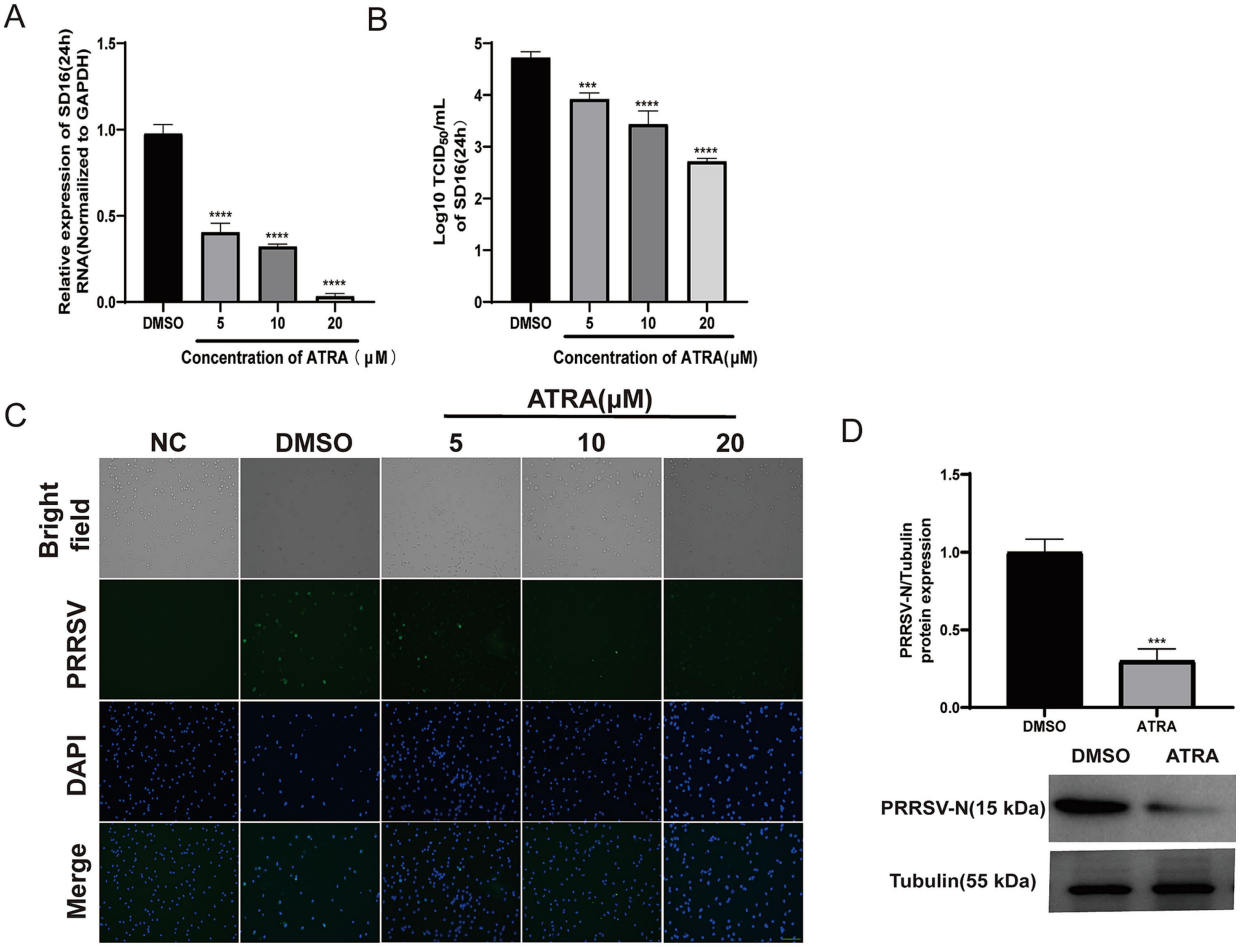
Retinoic acid inhibits highly pathogenic PRRSV infection. **(A)** RT-qPCR analysis of SD-16 RNA in PAMs treated with different concentrations of ATRA (24 h). DMSO was used as negative controls. Values represent mean ± SD, *n* = 3. **(B)** Viral titration analysis in TCID_50_/mL for SD-16 in supernatants of PAMs treated as described in **(A)**. Values represent mean ± SD, *n* = 3. **(C)** Representative immunofluorescence images of PAMs infected with or without SD-16 and treated with DMSO or ATRA at 24 h post-infection. NC: Non-infected cells treated with DMSO. DMSO: Infected cells treated with DMSO. ATRA: Infected cells treated with 5–20 μM ATRA. Blue represents DAPI and green represents PRRSV. Bar = 200 μM. ***p < 0.001, ****p < 0.0001. **(D)** The antiviral effect of ATRA was evaluated by WB analysis of SD-16N protein levels. (Top) The relative protein expression of PRRSV-N was analyzed by western blotting and normalized to the loading control tubulin. The bar graph shows the mean ± SD. ***p < 0.001, *n* = 3. (Bottom) Representative WB images of PRRSV-N (15 kDa) and tubulin (55 kDa).

We further tested if ATRA would inhibit the replication of the highly pathogenic (HP)-PRRSV variant – SD16 (23). PAMs were inoculated with SD16 virus, followed by the 5–20 μM ATRA gradient treatments. RT-qPCR revealed that at 24 hpi, the PRRSV RNA level was significantly reduced in an ATRA dosage-dependent manner (Figure 3A), which was further confirmed by the decreased viral titer of 0.6, 1, and 2 log at 5, 10, and 20 μM ATRA treatments, respectively (Figure 3B). Consistent with these data, immunofluorescence for the PRRSV N protein displayed an obvious reduction of infected PAMs by 5–20 μM ATRA treatments (Figure 3C). WB analysis was also performed, and the results showed that the protein level of PRRSV N was significantly reduced after treatment with 20 μM ATRA (Figure 3D). Thus, ATRA significantly inhibited the PRRSV infection of the primary targeting cells in pigs.

### Retinoic acid stimulates antiviral responses while suppressing unnecessary immune reactions

**Figure 4:**
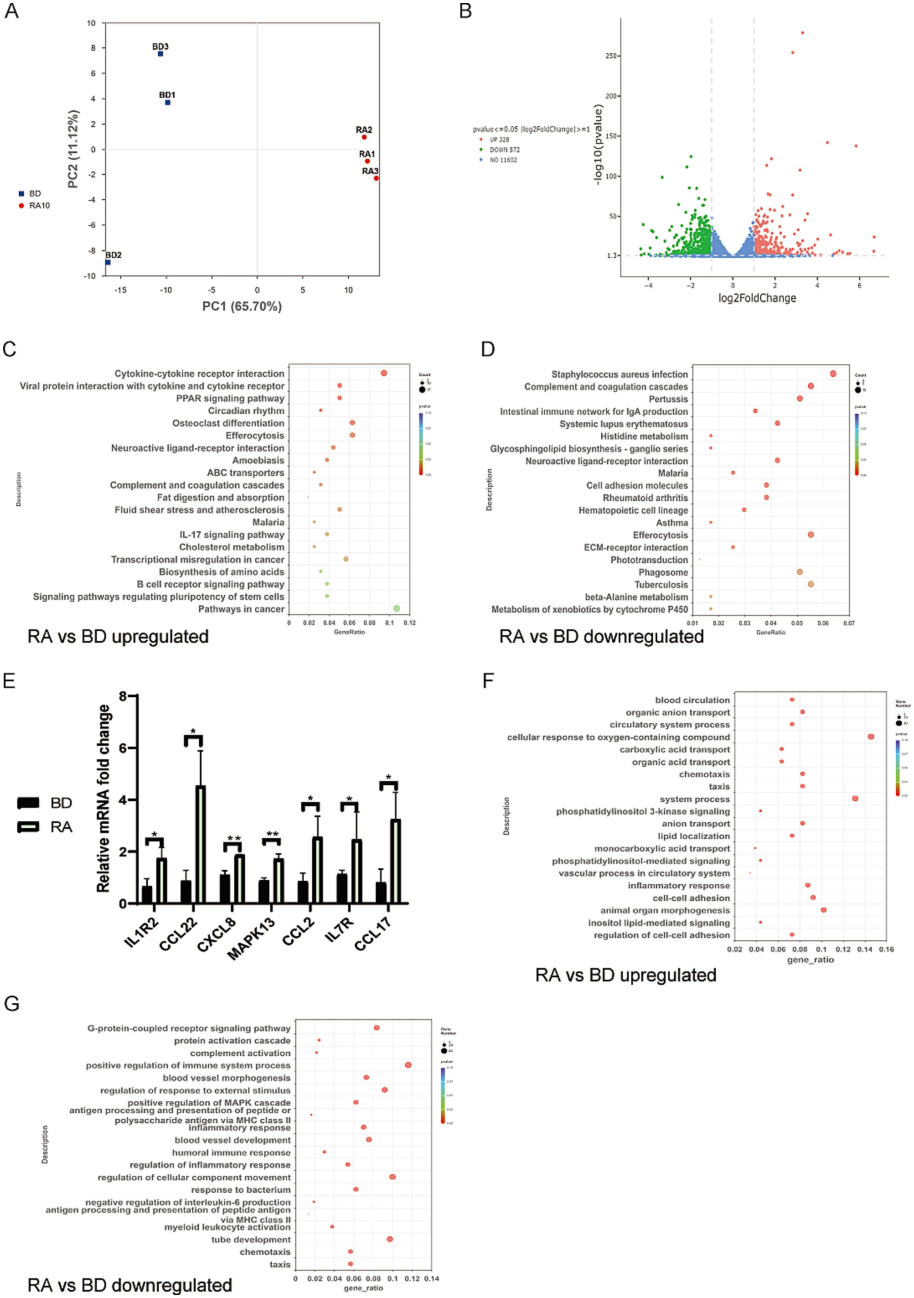
Retinoic acid stimulates antiviral responses while suppressing unnecessary immune interactions in PAMs. **(A)** PCA of RNA-seq data. BD: Non-infected cells treated with DMSO. RA: PAMs treated with 10 μM ATRA. **(B)** Volcano plot of differentially expressed genes (DEGs) in the RA group versus the BD group. **(C)** KEGG enrichment: upregulated DEGs (ATRA-treated vs. Control) from **(A)** RNA-seq data. **(D)** KEGG enrichment: downregulated DEGs (ATRA-treated vs. Control) from **(A)** RNA-seq data. **(E)** Relative mRNA expression of selected genes by RT-qPCR. Bar graph shows mean ± SD, *n* = 3. **(F)** Biological process of **(A)** RNA-seq DEGs: upregulated (ATRA-treated/Control). **(G)** Biological process of **(A)** RNA-seq DEGs: downregulated (ATRA-treated/Control).

To clarify the direct regulatory effects of ATRA on PAMs, we performed RNA-seq of PAMs treated with 10 μM ATRA for 24 h. Principal Component Analysis (PCA) showed a clear separation between the DMSO control and ATRA-treated groups (Figure 4A). Following ATRA treatment, we identified 328 upregulated and 572 downregulated differentially expressed genes (DEGs) compared with the control PAMs (Figure 4B). The KEGG analysis was performed, and the enriched KEGG pathways on upregulated DEGs primarily centered on foundational immune signaling, cellular homeostasis maintenance, and metabolic regulation (Figure 4C; Supplementary Table S1). The most significantly enriched pathways, Cytokine-cytokine receptor interaction, and viral protein interaction with cytokine and cytokine receptor were upregulated, along with the IL-17 signaling pathway; the enrichment of Effercytosis reinforces PAMs’ ability to clear apoptotic cells (24, 25). These data indicate enhanced immune responses against potential viral infection. The enrichment of the peroxisome proliferator-activated receptor (PPAR) signaling pathway reaffirms the role of RA-signaling in maintaining basal lipid metabolic homeostasis (26).

In contrast, the downregulated KEGG pathways focused on excessive immune activation and non-specific pathogen responses (Figure 4D; Supplementary Table S2): *Staphylococcus aureus* infection and Pertussis were suppressed; Complement and coagulation cascades and Cell adhesion molecules were also downregulated. These reduce unnecessary immune resource consumption in quiescent (naïve) PAMs. Additionally, Systemic lupus erythematosus downregulation reinforced RA’s role in maintaining immune homeostasis, reducing the risk of autoimmunity-related aberrant responses in PAMs.

To validate the transcriptomic sequencing data, we performed RT-qPCR analysis on seven key genes selected from the upregulated Cytokine-cytokine receptor interaction, and Viral protein interaction with cytokine and cytokine receptor, and the IL-17 signaling pathways upon ATRA-treatment. The expression patterns of these genes were consistent with the transcriptomic results (Figure 4E), confirming the results of our RNA-seq analysis.

GO analysis on the upregulated DEGs also revealed significant enrichment in immune-inflammatory-related biological processes (BPs), including Chemotaxis, Taxis, and Inflammatory response (Figure 4F; Supplementary Table S3). Other upregulated GO terms were primarily associated with metabolic transport, cellular signaling, and environmental response processes, including carboxylic acid transport, organic acid transport, organic anion transport, phosphatidylinositol 3-kinase signaling pathway, and cellular response to oxygen-containing compounds. In contrast, GO analysis on the downregulated DEGs were concentrated in immune activation, inflammatory response, signal transduction, and vascular-related processes (Figure 4G; Supplementary Table S4). These include Inflammatory response, Antigen processing and presentation of peptide or polysaccharide antigen via major histocompatibility complex (MHC) class II, Complement activation, Positive regulation of immune system process, Positive regulation of mitogen-activated protein kinase (MAPK) cascade, G-protein-coupled receptor signaling pathway, Blood vessel and morphogenesis, etc.

Overall, the KEGG analysis results synergistically corroborate the GO findings, indicate that RA signaling exerts a basal immune homeostasis-regulatory role in PAMs, reducing unnecessary signal perturbations by inhibiting excessive immune activation, while stimulating antiviral cytokine responses in quiescent cells.

### Transcriptomics analysis uncovers molecular regulatory profiles by ATRA treatment in PRRSV infection

**Figure 5:**
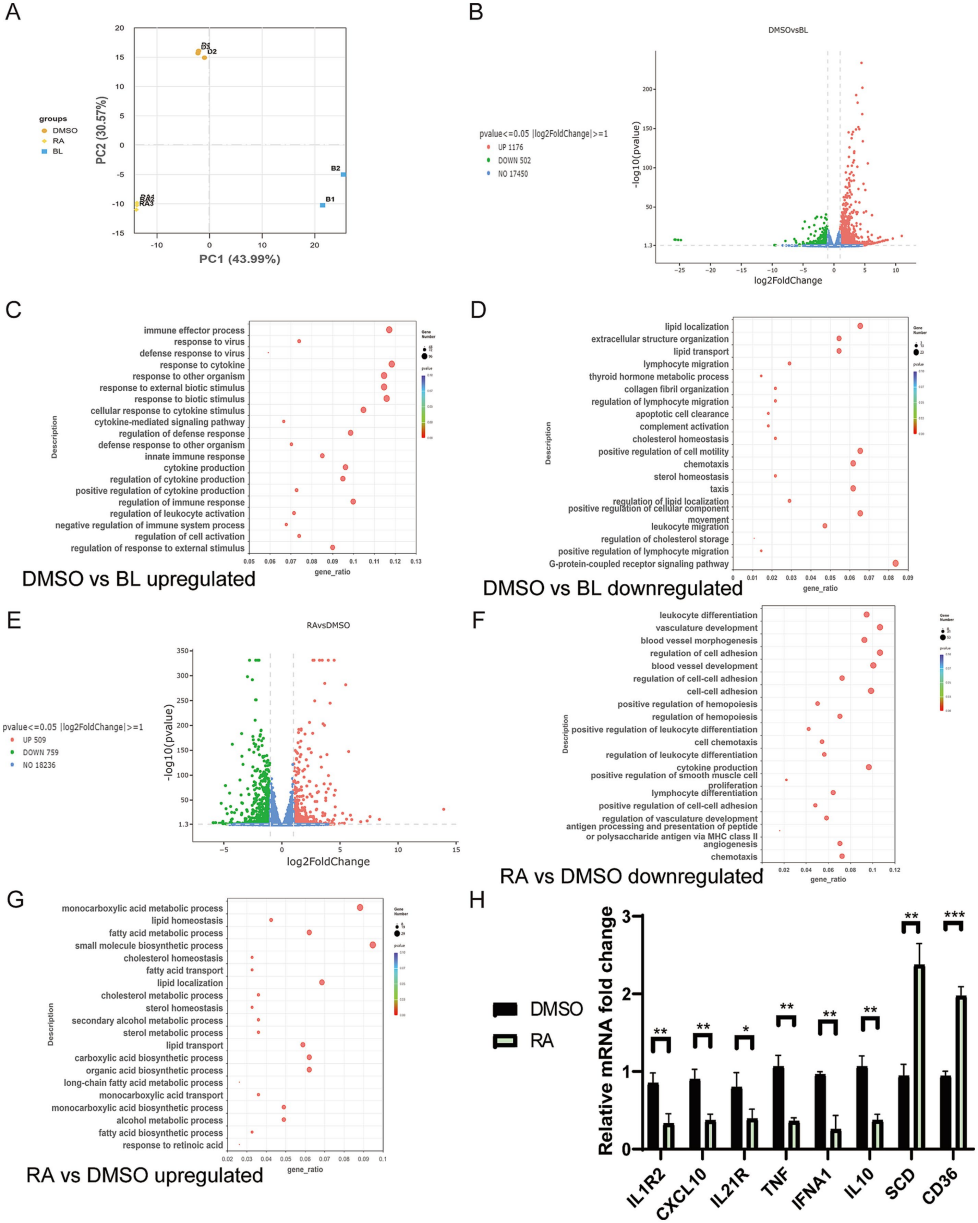
Transcriptomic analysis uncovers molecular regulatory profiles of PRRSV infection and retinoic acid treatment in PAMs. **(A)** PCA of RNA-seq data. BL: Non-infected cells treated with DMSO. DMSO: PRRSV VR2332 infected cells treated with DMSO. RA: PRRSV VR2332-infected cells treated with 10 μM ATRA. **(B)** Volcano plot of differentially expressed genes (DEGs) in the DMSO group versus the BL group. **(C)** Biological process of **(A)** RNA-seq DEGs: Upregulated (PRRSV infected/Ctrl). **(D)** Biological process of **(A)** RNA-seq DEGs: Downregulated (PRRSV infected/Ctrl). **(E)** Volcano plot of differentially expressed genes (DEGs) in the RA group versus the DMSO group. **(F)** Biological process of **(A)** RNA-seq DEGs: Downregulated (ATRA-treated/PRRSV infected). **(G)** Biological process of **(A)** RNA-seq DEGs: Upregulated (ATRA-treated/PRRSV infected). **(H)** Relative mRNA expression of selected genes by RT-qPCR. Bar graph shows mean ± SD, *n* = 3.

To investigate the underlying mechanism of RA inhibition of PRRSV infection, we performed RNA-seq of uninfected PAMs and PAMs infected with PRRSV with or without 10 μM ATRA treatment for 24 h. Principal Component Analysis (PCA) showed a clear separation between the DMSO control and PRRSV-infected groups along both PC1 and PC2 axes, while the RA-treated group exhibited a tendency of correction along the PC2 axis (Figure 5A). Following PRRSV infection, we identified 1,176 upregulated and 502 downregulated DEGs compared with the control PAMs (Figure 5B). Gene Ontology (GO) analysis identified that the upregulated DEGs were significantly enriched in immune-inflammatory and antiviral response-related BPs (Figure 5C; Supplementary Table S5). These include Response to external stimulus, Defense response to virus; Response to virus, Immune effector process, and Cytokine-mediated signaling pathway, demonstrating a strong immune response initiated by PAMs to the PRRSV infection.

For the downregulated DEGs upon PRRSV infection, GO analysis indicated that PRRSV infection mediates immune evasion and disrupts cellular metabolic functions (Figure 5D; Supplementary Table S6). Immune regulatory pathways such as G protein-coupled receptor signaling pathway, Positive regulation of lymphocyte migration, Complement activation, as well as metabolic homeostasis pathways including Regulation of lipid localization and Steroid hormone homeostasis were significantly downregulated (Figure 5D). Downregulation of the complement activation pathway—a central cascade for innate immune clearance of pathogens (27)—suggests that PRRSV achieves immune evasion by impairing the early phagocytic clearance capacity of PAMs, consistent with the role of PAMs as the primary replication niche for PRRSV.

Compared with the PRRSV-infected cells, there are 509 upregulated and 759 downregulated DEGs in ATRA-treated, PRRSV infected PAMs (Figure 5E). For the downregulated DEGs, GO analysis on ATRA treatment showed enrichment on overactivated immune processes, such as Peptide/polysaccharide antigen presentation, Lymphocyte differentiation and leukocyte chemotaxis (Figure 5F; Supplementary Table S7). Notably, there is downregulation of the MHC class II antigen presentation pathway, which is consistent with the findings in ATRA-treated uninfected PAMs (Figure 4F), implying that ATRA may dampen adaptive immune initiation to mitigate excessive immune-mediated injury. On the other hand, GO analysis on ATRA treatment revealed specifically upregulated metabolic pathways, including Response to retinoic acid (as expected) and Fatty acid biosynthetic process (Figure 5G; Supplementary Table S8). Importantly, the RA-upregulated pathways such as Lipid transport and Lipid localization precisely corresponded to the same metabolic homeostasis pathways suppressed by PRRSV infection (Figures 5D,G).

Consistent with GO enrichment results, comparison of overlapping KEGG pathways upregulated in PRRSV-infected PAMs and downregulated in ATRA-treated, PRRSV-infected PAMs also revealed that ATRA exerts anti-PRRSV effects through targeted suppression of core PRRSV-activated inflammatory and immune regulatory pathways (Supplementary Figures S2, S3; Supplementary Tables S9, S10). Specifically, the Cytokine-cytokine receptor interaction pathway was significantly upregulated in PRRSV-infected PAMs. The excessive activation of this pathway drives a cytokine storm not only constructing a favorable inflammatory microenvironment for viral replication but also exacerbates pulmonary immunopathological damage (28). Notably, this pathway was significantly downregulated following ATRA treatment, directly blocking the cascade amplification of PRRSV-induced cytokine signaling. Concurrently, the Th17 cell differentiation pathway, which was upregulated upon PRRSV infection, was synchronously suppressed by ATRA. Aberrant activation of Th17 cell differentiation leads to biased imbalance of adaptive immune responses and excessive inflammatory reactions that aggravate tissue damage (29). Furthermore, the inflammatory bowel disease pathway was significantly upregulated post-infection—reflecting the tendency of inflammatory responses to spread systemically following viral infection. ATRA treatment downregulated this pathway, effectively mitigating infection-mediated systemic inflammatory responses and auxiliary enhancing the host’s tolerance to infection. KEGG analysis on the downregulated DEGs post-PRRSV infection also revealed primary enrichment in innate immune and metabolic homeostasis-related pathways (Supplementary Figure S4; Supplementary Table S11). Specifically, the Complement and coagulation cascades and PPAR signaling pathway exhibited relatively high enrichment significance. Downregulation of the PPAR signaling pathway exacerbates PAM lipid accumulation (30, 31), a critical microenvironment for PRRSV replication. ATRA specifically upregulated metabolic pathways such as the PPAR signaling pathway and Fatty acid metabolism pathway (Supplementary Figure S5; Supplementary Table S12). RT-qPCR analysis of eight key pathway genes (TNF, IL1R2, CD36, IFNA1, IL10, IL21R, FASN and CXCL10) was consistent with the transcriptomic data (Figure 5H). These genes are associated with the upregulated pathways (PPAR signaling pathway-CD36, fatty acid metabolism-SCD) and downregulated pathways (cytokine-cytokine receptor interaction-TNF, IL10, IL1R2, viral protein interaction with cytokine and cytokine receptor-TNF, IL10, CXCL10 and Th17 cell differentiation-IL21R) upon ATRA treatment, further validating the RNA-seq results.

Collectively, integrating KEGG and GO results reveals that the RA-signal shapes a well-equipped basal function and moderate immune activation homeostatic phenotype in PAMs. It enhances foundational immune signaling, metabolic support, and environmental adaptation while curbing excessive immune activation and non-specific pathogen responses. This regulatory pattern reserves functional potential for precise immune initiation and controllable inflammatory damage during subsequent pathogen infection, which functionally aligned with ATRA’s anti-inflammatory and metabolic repair effects observed in the PRRSV infected PAMs.

## Discussion

Our study demonstrates that ATRA, the major biologically active form of RA in mammalian immune cells, effectively inhibits PRRSV infection in both permissive cell lines and the primary target cells - PAMs in pigs, including highly pathogenic strains without significant cytotoxicity. These findings establish RA (via its canonically active form ATRA) as a potent antiviral agent against PRRSV and provide mechanistic insights into its mode of action through transcriptomic profiling.

PRRSV is notorious for its ability to subvert host immune responses, particularly by suppressing interferon production and promoting inflammatory cytokine release (32), which together create a favorable environment for viral replication and exacerbate lung immunopathology. RA has been previously shown to enhance type I IFN responses via IRF3 and CaMKK activation, suggesting its potential to counteract PRRSV-mediated IFN suppression (17). This inhibition effectively impairs the inflammatory microenvironment dependent on viral replication and alleviates infection-mediated lung tissue injury, making it the core target pathway for ATRA’s anti-PRRSV effects; Studies have shown that the immunomodulatory effect of ATRA is reflected in two aspects: first, it inhibits RORγt, a key transcription factor of T helper 17 cells (Th17); second, it promotes the expression of Foxp3, a key transcription factor of regulatory T cells (Treg) (33). Consistent with this notion, our RNA-seq data indicated that ATRA treatment downregulates several hyperactivated immune pathways induced by PRRSV infection, including cytokine-cytokine receptor interaction and Th17 cell differentiation, which aligned with the established immunomodulatory roles of RA and likely contributed to attenuating excessive inflammation during PRRSV infection (7, 34).

Beyond immune modulation, our pathway analyses reveal that PRRSV infection significantly disrupts metabolic homeostasis in PAMs, particularly lipid metabolism pathways such as the PPAR signaling. This disruption may promote intracellular lipid accumulation, which has been linked to enhanced PRRSV replication (35). Notably, ATRA treatment restored the PPAR signaling and fatty acid metabolic processes, suggesting that RA not only curbs inflammatory damage but also rectifies virus-induced metabolic dysfunction, thereby depriving PRRSV of a conducive replication niche.

In terms of pathway expression characteristics, the upregulation trends of the cytokine-cytokine receptor interaction pathway and the viral protein interaction with cytokines and cytokine receptors pathway in ATRA treated, non-infected PAMs are consistent with the upregulation patterns of these pathways in PRRSV-infected groups. Meanwhile, the downregulation trends of the Cell adhesion molecules, Hematopoietic cell lineage, extracellular matrix (ECM)-receptor interaction, and Complement and coagulation cascades pathways in ATRA treated non-infected PAMs are also consistent with the expression change directions of these pathways in PRRSV-infected groups. These pathway-level expression correlation characteristics may explain why, in the PCA analysis along the PC1 axis, samples from the RA-treated group exhibit a distribution pattern that is more closely aligned with that of PRRSV-infected group samples. Nevertheless, these findings imply that ATRA treatment primes the immune responses in PAMs to a level that is similar to in real pathogen infection, which ensures effective viral clearance upon PRRSV infection.

Collectively, ATRA exerts its antiviral activity through the multi-dimensional synergistic inhibition of PRRSV-activated pathways that mediate cytokine signaling, adaptive immune differentiation and systemic inflammation, consequently impairing viral replication niches and maintaining host immune homeostasis. Together, these data delineate the molecular perturbation characteristics of PRRSV-infected PAMs and identify the core pathway targets through which ATRA modulates PAM functions at both immune and metabolic levels, providing a solid molecular basis for subsequent functional validations and investigations into new intervention strategies against PRRSV infection.

Building upon this molecular basis, it is important to contextualize the main strengths and limitations of our study. The primary strength of this work lies in the comprehensive evaluation of ATRA’s antiviral efficacy using not only MARC-145 cell lines but also primary PAMs, which accurately represent the natural target cells of PRRSV *in vivo*. Coupled with transcriptomic profiling, this physiologically relevant approach allowed us to confidently uncover a novel dual mechanism: ATRA attenuates virus-induced hyperinflammatory responses while simultaneously restoring lipid metabolic homeostasis. Nevertheless, the current study is inherently limited by its reliance on *in vitro* and ex vivo models. To translate these findings into clinical veterinary practice, future in vivo animal studies are indispensable for assessing the pharmacokinetics, systemic safety, and actual protective efficacy of ATRA in PRRSV-challenged pigs. Additionally, while our RNA-seq data successfully highlight key pathways like PPAR signaling, definitive validation of these precise molecular targets will require further studies employing specific receptor antagonists or targeted gene-editing techniques. Acknowledging these limitations, our findings still provide a robust scientific rationale for pursuing retinoic acid as a host-directed therapeutic option. In conclusion, our work establishes that ATRA inhibits PRRSV infection through a multifaceted mechanism involving suppression of hyperinflammatory responses, restoration of metabolic homeostasis, and modulation of innate and adaptive immune pathways. These findings position ATRA as a promising candidate for developing adjunctive immunomodulatory strategies against PRRS, particularly in mitigating virus-induced immunopathology and improving the host resistance.

## Data Availability

All RNA-seq data are stored in the Genome Sequence Archive (GSA) of China National Center for Bioinformation (CNCB) under accession number PRJCA055876. All other data generated during the current study are included in this manuscript.

## Supplementary material

SUPPLEMENTARY FIGURE S1 Flow cytometry scatter plots: PRRSV-GFP infection rate (BL1 H:GFP-H, x-axis) and cell viability (YL1-H-PI-H, y-axis) in cells treated with DMSO or different ATRA concentrations.

SUPPLEMENTARY FIGURE S2 KEGG enrichment: upregulated DEGs (infected/control).

SUPPLEMENTARY FIGURE S3 KEGG enrichment: downregulated (ATRA-treated/control).

SUPPLEMENTARY FIGURE S4 Downregulated DEGs (infected/control).

SUPPLEMENTARY FIGURE S5 Upregulated DEGs (ATRA–treated/control).

SUPPLEMENTARY TABLE S1 KEGG enrichment analysis of upregulated genes: ATRA-treated versus DMSO-treated groups (PRRSV-Uninfected).

SUPPLEMENTARY TABLE S2 KEGG enrichment analysis of downregulated genes: ATRA-treated versus DMSO-treated groups (PRRSV-Uninfected).

SUPPLEMENTARY TABLE S3 GO enrichment analysis of upregulated genes: ATRA-treated versus DMSO-treated groups (PRRSV-Uninfected).

SUPPLEMENTARY TABLE S4 GO enrichment analysis of downregulated genes: ATRA-treated versus DMSO-treated groups (PRRSV-Uninfected).

SUPPLEMENTARY TABLE S5 GO enrichment analysis of upregulated genes: PRRSV-infected versus DMSO-treated control group.

SUPPLEMENTARY TABLE S6 GO enrichment analysis of downregulated genes: PRRSV-infected versus DMSO-treated control group.

SUPPLEMENTARY TABLE S7 GO enrichment analysis of downregulated genes in PRRSV infected cells: ATRA-treated versus DMSO-treated groups.

SUPPLEMENTARY TABLE S8 GO enrichment analysis of upregulated genes in PRRSV-infected cells: ATRA-treated versus DMSO-treated groups.

SUPPLEMENTARY TABLE S9 KEGG enrichment analysis of upregulated genes: PRRSV-infected versus DMSO-treated control group.

SUPPLEMENTARY TABLE S10 KEGG enrichment analysis of downregulated genes in PRRSV infected cells: ATRA-treated versus DMSO-treated groups.

SUPPLEMENTARY TABLE S11 KEGG enrichment analysis of downregulated genes: PRRSV infected versus DMSO-treated control group.

SUPPLEMENTARY TABLE S12 KEGG enrichment analysis of upregulated genes in PRRSV infected cells: ATRA-treated versus DMSO-treated groups.
