# Supplementary figures and images for: Retinoic acid inhibits the infection of porcine reproductive and respiratory syndrome virus
Source: Front Vet Sci. 2026 Mar 24;13:1798441. doi: 10.3389/fvets.2026.1798441 (PMC13053225; doi:10.3389/fvets.2026.1798441)

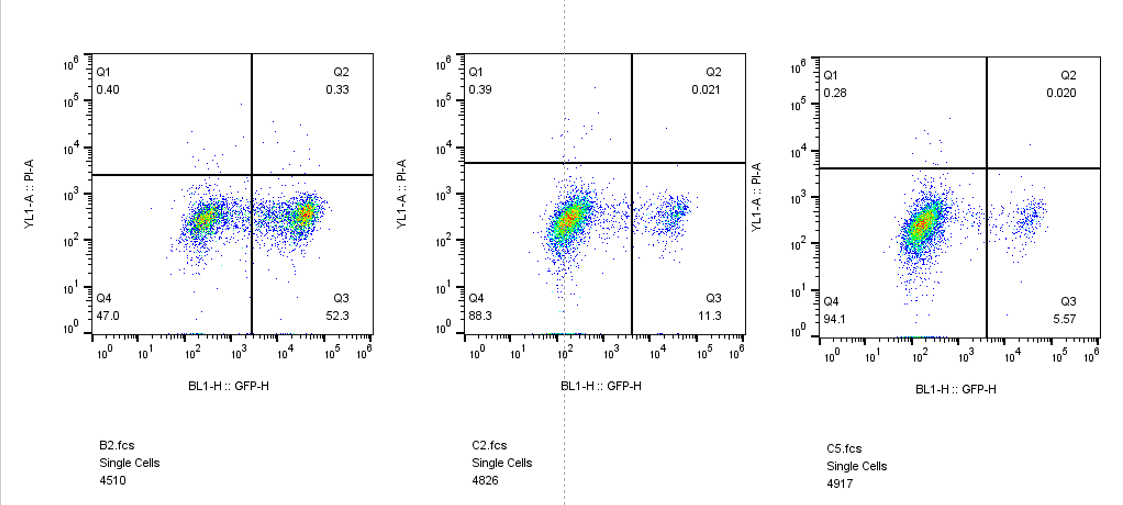

Supplement: SUPPLEMENTARY FIGURE S1 — Flow cytometry scatter plots: PRRSV-GFP infection rate (BL1 H:GFP-H, x-axis) and cell viability (YL1-H-PI-H, y-axis) in cells treated with DMSO or different ATRA concentrations. [file Image_1.PNG]

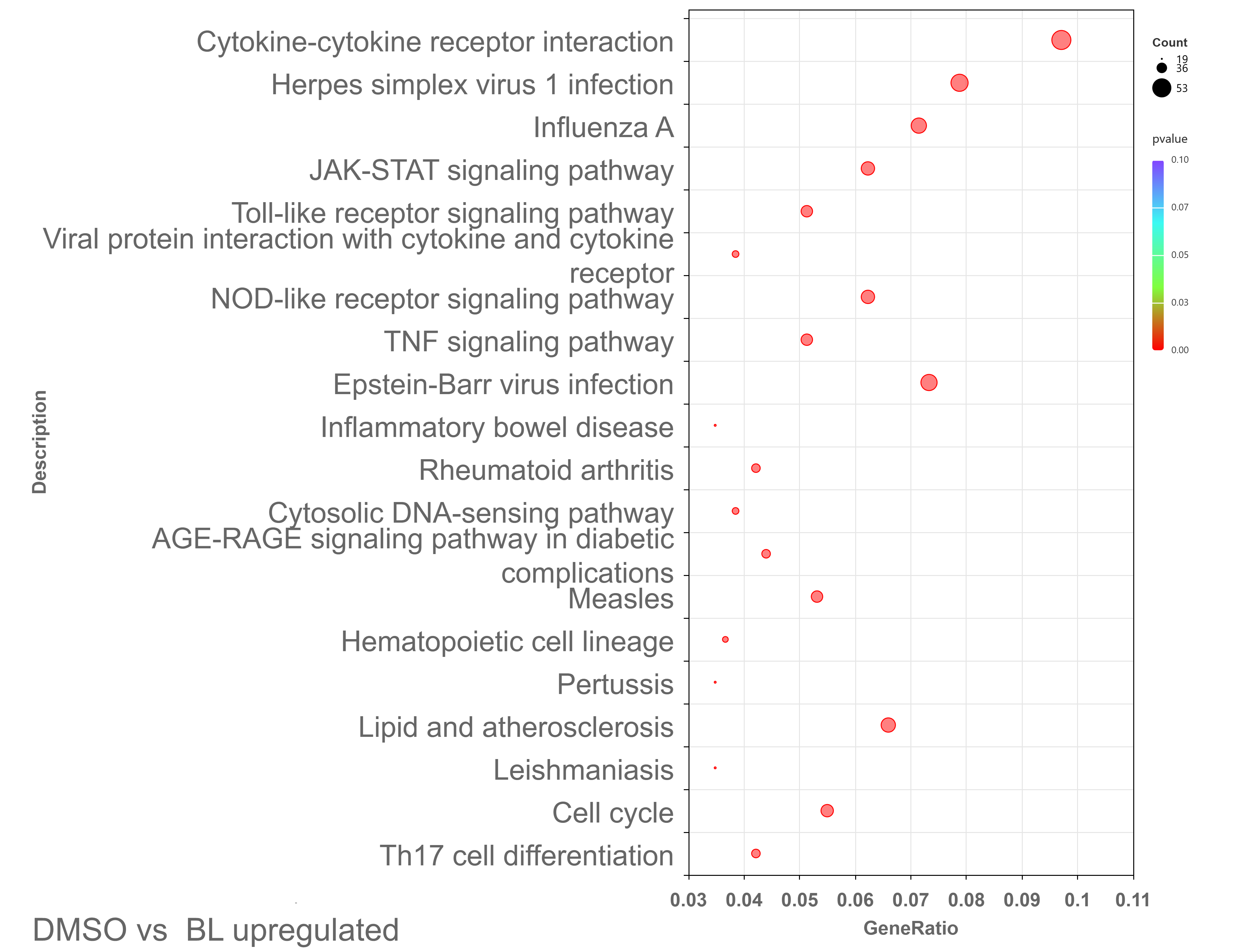

Supplement: SUPPLEMENTARY FIGURE S2 — KEGG enrichment: upregulated DEGs (infected/control). [file Image_2.TIFF]

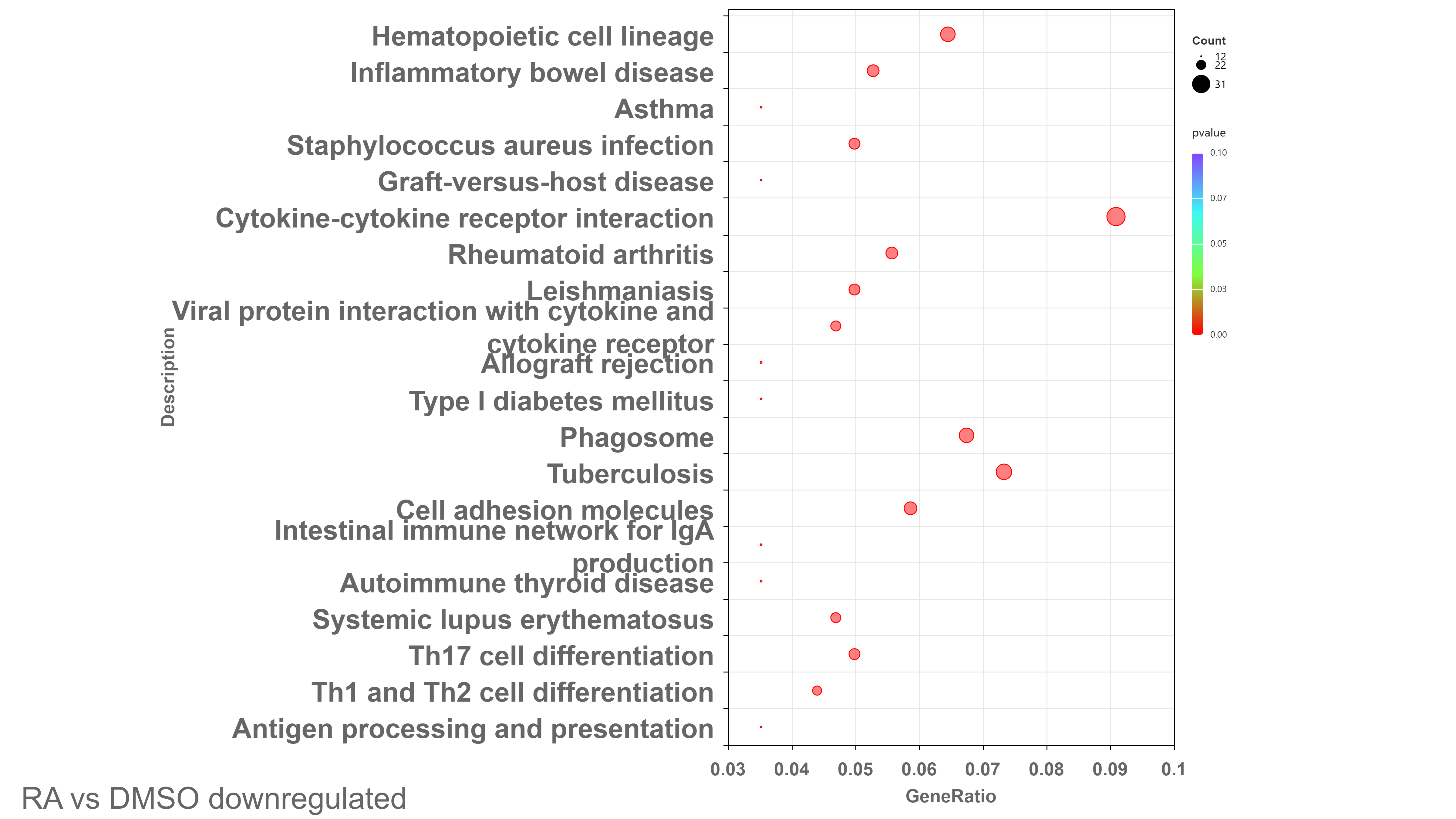

Supplement: SUPPLEMENTARY FIGURE S3 — KEGG enrichment: downregulated (ATRA-treated/control). [file Image_3.TIF]

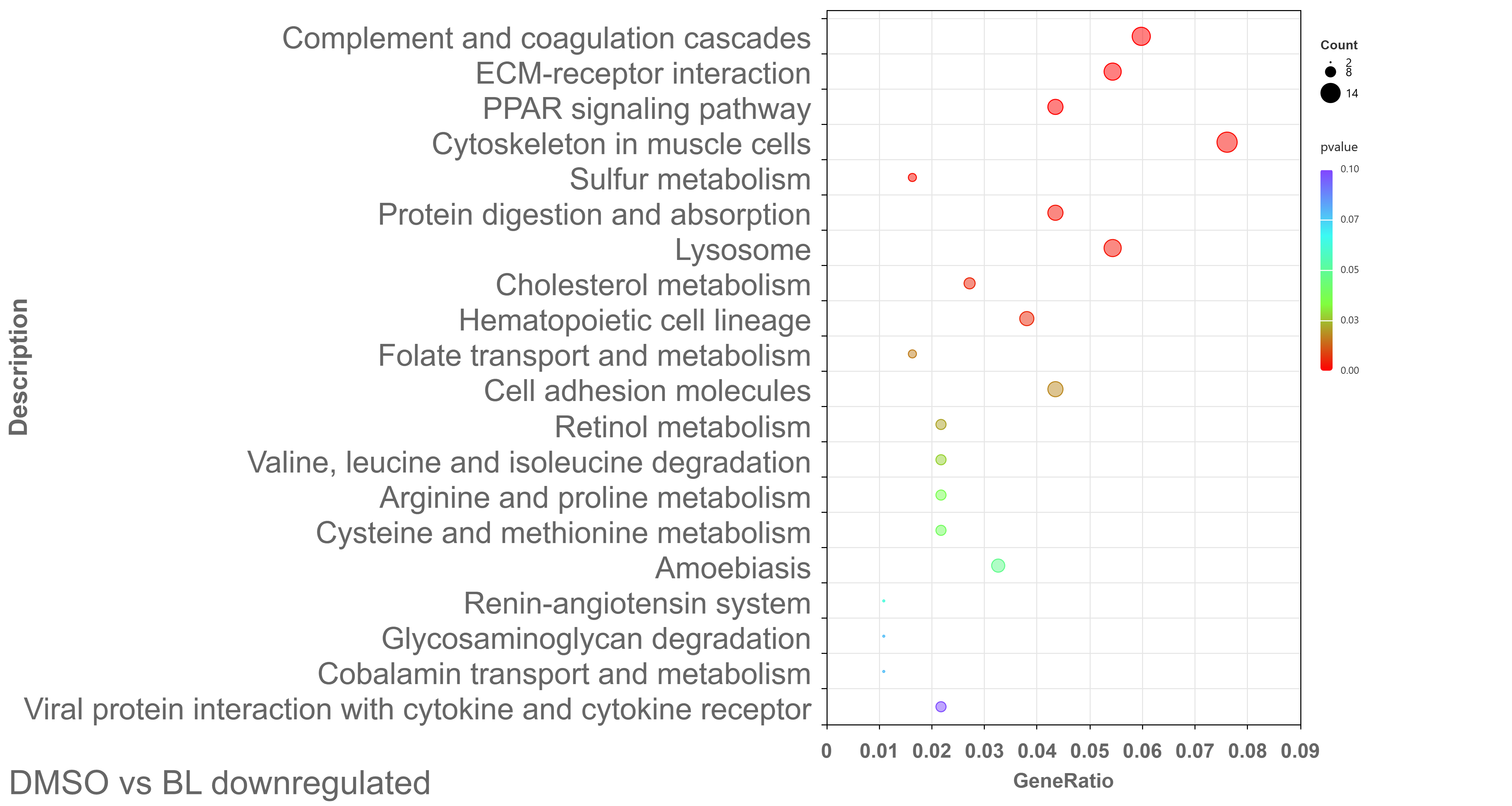

Supplement: SUPPLEMENTARY FIGURE S4 — Downregulated DEGs (infected/control). [file Image_4.TIF]

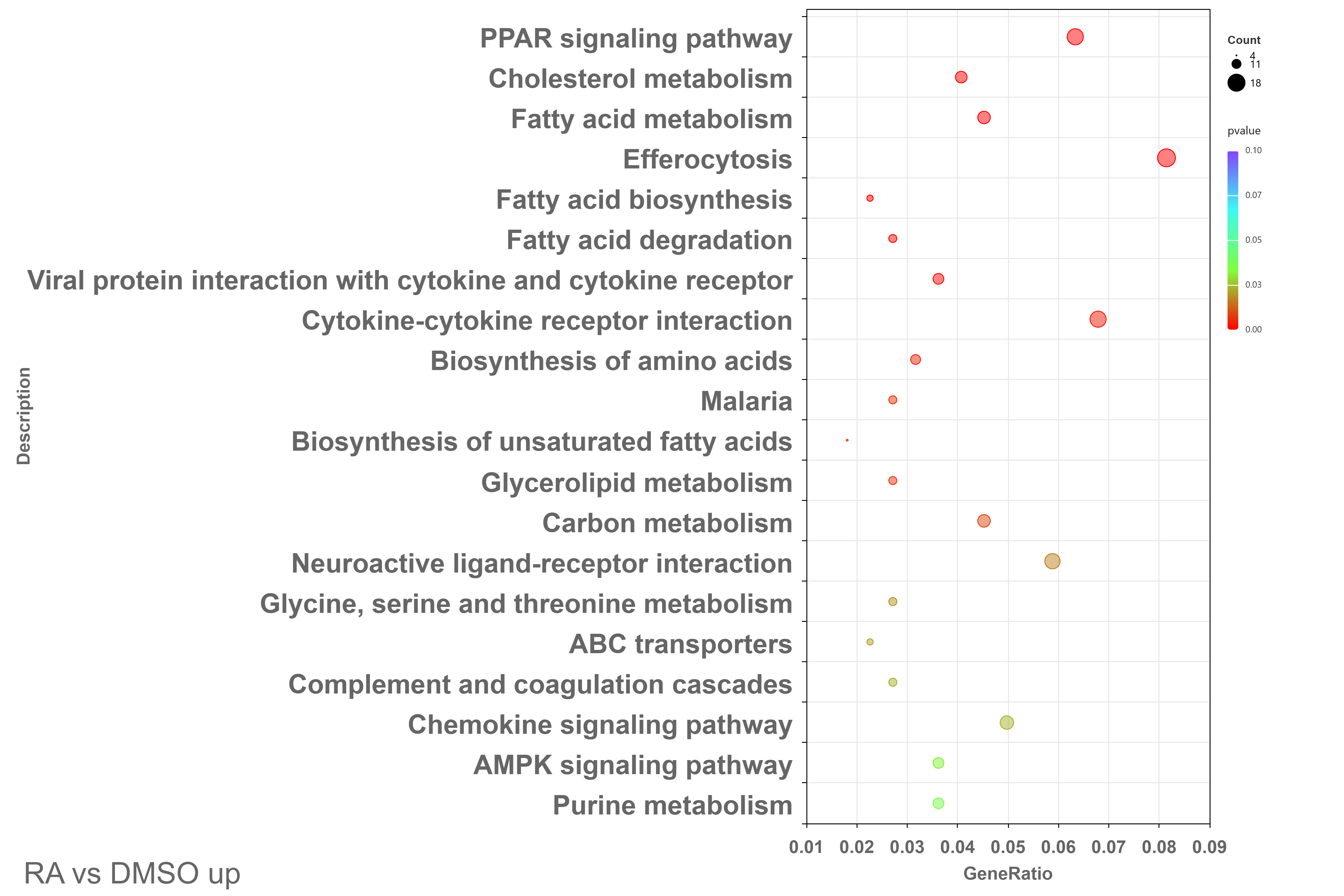

Supplement: SUPPLEMENTARY FIGURE S5 — Upregulated DEGs (ATRA–treated/control). [file Image_5.TIF]
