# Supplementary material for: Retinoic acid inhibits the infection of porcine reproductive and respiratory syndrome virus
Source: Front Vet Sci. 2026 Mar 24;13:1798441. doi: 10.3389/fvets.2026.1798441 (PMC13053225; doi:10.3389/fvets.2026.1798441)
Supplement: Supplementary file 18 [file Table_13.DOCX]

**Table S13. Primers for RT-qPCR**

| **RT-qPCR Primers Table** | | |  |  |  |
| --- | --- | --- | --- | --- | --- |
| **Target** |  | **Sequence (5’ → 3’)** |  |  |  |
| PRRSV-  VR | Forward | AAACCAGTCCAGAGGCAAGG |  |  |  |
|  | Reverse | GCAAACTAAACTCCACAGTGTAA |  |  |  |
| PRRSV-  SD16 | Forward | AAACCAGTCCAGAGGCAAGG |  |  |  |
|  | Reverse | GCAAACTAAACTCCACAGTGTAA |  |  |  |
| pGAPDH | Forward | CATCCTGGGCTACACTGAGG |  |  |  |
|  | Reverse | GCTTGACGAAGTGGTCGTTG |  | Forward | AAACCAGTCCAGAGGCAAGG |
| pIL7R | Forward | GGTACCACTCTTGGTATGGCT |  | Reverse | GCAAACTAAACTCCACAGTGTAA |
|  | Reverse | TGCGTCGTCAAAGTCTCCATT |  | Forward | AAACCAGTCCAGAGGCAAGG |
| pCXCL8 | Forward | TGGACCCCAAGGAAAAGTGG |  | Reverse | GCAAACTAAACTCCACAGTGTAA |
|  | Reverse | TGTTGTTGCTTCTCAGTTCTC |  | Forward | CATCCTGGGCTACACTGAGG |
| pIL1R2 | Forward | ACGGTGCTCTCTGGATTGTG |  | Reverse | GCTTGACGAAGTGGTCGTTG |
|  | Reverse | TCAGTCTCGAGCCCAGTGAA |  | Forward | ACGGTGCTCTCTGGATTGTG |
| pCCL22 | Forward | GCGTGGTCCTGCTAACCTTG |  | Forward | ACGGTGCTCTCTGGATTGTG |
|  | Reverse | AATCTTCTTCACCCAGGGCAG |  | Forward | GCGTGGTCCTGCTAACCTTG |
| pCD36 | Forward | TGGGCTGCAATAGAGACTGTG |  | Forward | GCGTGGTCCTGCTAACCTTG |
|  | Reverse | ATACCTCCAAACACGGCCAG |  | Forward | TGGGCTGCAATAGAGACTGTG |
| pMAPK13 | Forward | CCTGCGTAACTTCCACGACT |  | Forward | TGGGCTGCAATAGAGACTGTG |
|  | Reverse | CCAGCGGAGTGGATGTACTTT |  | Forward | TGGGCTGCAATAGAGACTGTG |
| pCCL17 | Forward | CAGGGACGCCATTGTGCTTG |  | Forward | TGGGCTGCAATAGAGACTGTG |
|  | Reverse | ACAGGGTCCTGTGGCTTCAT |  | Forward | CAGGGACGCCATTGTGCTTG |
| pCCL2 | Forward | GAACCCAAGCAGAAGTGGGT |  | Forward | CAGGGACGCCATTGTGCTTG |
|  | Reverse | CAAGGCTTCGGAGTTTGGTTT |  | Forward | GAACCCAAGCAGAAGTGGGT |
| pIFNA1 | Forward | TATCTGCAAGAGAAGAGCTACAGCC |  | Forward | GAACCCAAGCAGAAGTGGGT |
|  | Reverse | TCATGACTTCTGCCCTGACGA |  | Forward | TATCTGCAAGAGAAGAGCTACAGCC |
| pTNF | Forward | TATCGGCCCCCAGAAGGAAG |  | Forward | TATCTGCAAGAGAAGAGCTACAGCC |
|  | Reverse | CGGCTTTGACATTGGCTACAAC |  | Forward | TATCGGCCCCCAGAAGGAAG |
| pIL10 | Forward | TGCGGCGCTGTCATCAAT |  | Forward | TATCGGCCCCCAGAAGGAAG |
|  | Reverse | GGCTTTGTAGACACCCCTCTC |  | Forward | TGCGGCGCTGTCATCAAT |
| pSCD | Forward | CTTCGTTACGCCATCGTGCT |  | Forward | TGCGGCGCTGTCATCAAT |
|  | Reverse | GTGGAAGCCCTCACCCACAG |  | Forward | GGCTTCTAGAGGCTTCCCAC |
| pCXCL10 | Forward | CCAGAACTGTTCGCTGTACCT |  | Forward | GGCTTCTAGAGGCTTCCCAC |
|  | Reverse | GGGCAAGATTGACTTGCAGGA |  | Reverse | TGGTACACCTTCCCGCTTG |
| pIL21R | Forward | CACCCTCACCTGGCAAGAC |  |  |  |
|  | Reverse | TGTCGTCAGCCATGAATTGGA |  |  |  |
